# Is CCR5-Δ32 mutation associated with diabetic nephropathy in type 2 diabetes?

**DOI:** 10.4103/0256-4947.55177

**Published:** 2009

**Authors:** Mohammad K. Arababadi, Nima Naghavi, Gholamhossein Hassanshahi, Mehdi Mahmoodi

**Affiliations:** From the Rafsanjan University of Medical Sciences, Rafsanjan, Iran

**To the Editor:** Immunological factors like the chemokine-receptor axis recently were proved to have crucial roles in diabetes and its complications.^1^ CCR5 is a G-protein CC (CCL3, CCL4, CCL5) chemokine receptor, which recruits immune cells to the site of infection, inflammation and injuries, including nephropathic disease.^2^ Previous studies demonstrated that a mutation in the unique exon (exon 1) of the CCR5 and deletion of 32 nucleotides (also known as Δ32) leads to decreased expression and function of CCR5.^2^ Due to its immunomodulatory functions, the CCR5-Δ32 mutation seems to play a key role in autoimmune and inflammatory diseases.^3^ This study aimed to analyze the CCR5-Δ32 mutation in type 2 diabetic patients with and without diabetic nephropathy (DN). Samples were collected from 100 type 2 diabetic patients with DN, 200 type 2 diabetic patients without DN and 300 healthy controls. The groups were of the same sex, roughly the same age, diabetes history and socioeconomic status. DNA extraction and Gap-PCR reactions were performed as our previous study.^4^ Our findings indicated the absence of CCR5-Δ32 in type 2 diabetic patients with and without DN, while only two controls showed a heterozygotic pattern of mutation. A significant difference was not observed in the CCR5-Δ32 mutation in diabetic patients and controls (*P* <.78).

The CCR5 affects the immune cell function,^1^ and thus, the CCR5-Δ32 mutation leads to decreased CCR5 expression and influences the function of these cells.^3^ In agreement with our results, Kalev and colleagues reported a lack of association between CCR5-Δ32 mutation and DN in native Estonian type 2 diabetic patients.^2^ Studies also demonstrated that type 1 diabetes is not related to CCR5-Δ32.^5^,^6^ Like other investigators, we could not find any association between development of DN and CCR5-Δ32 mutation. Based on our findings and others, it seems that CCR5-Δ32 in diabetic type 2 patients is independent of geographical and ethnic factors. Prasad et al, showed that CCR5-Δ32 is not related to nephropatic type 2 diabetes in Asian-Indians.^7^ Another study in type 1 diabetic patients with renal failure showed that male carriers with CCR5-Δ32 mutation had a higher risk of nephropathy.^8^ To our knowledge none of research groups showed an association of CCR5-Δ32 with nephropathy in type 2 diabetic patients, but other CCR5 gene polymorphisms are associated with these nephropathies.^7^,^9^ Overall, based on the results of this study and globally collected data, it is likely that the CCR5-Δ32 mutation is not associated with diabetes and the nephropathic complications of diabetes.
